# Different Expression of Vascularization and Inflammatory Regulators in Cells Derived from Oral Mucosa and Limbus

**DOI:** 10.3390/bioengineering12070688

**Published:** 2025-06-24

**Authors:** Eleni Voukali, Joao Victor Cabral, Natalia Smorodinova, Vojtech Kolin, Magdalena Netukova, Tomáš Vacík, Katerina Jirsova

**Affiliations:** 1 Institute of Biology and Medical Genetics, First Faculty of Medicine, Charles University and General University Hospital in Prague, 128 00 Prague, Czech Republic; eleni.voukali@lf1.cuni.cz (E.V.); jvcabralmed@gmail.com (J.V.C.); natalia.smorodinova@lf1.cuni.cz (N.S.); tomas.vacik@lf1.cuni.cz (T.V.); 2 Department of Pathology of the Third Medical Faculty, University Hospital Kralovske Vinohrady, 100 34 Prague, Czech Republic; vojtech.kolin@lf3.cuni.cz; 3 Ophthalmology Department of the Third Medical Faculty, University Hospital Kralovske Vinohrady, 100 34 Prague, Czech Republic; magdalena.netukova@lf3.cuni.cz

**Keywords:** Limbal stem cell deficiency, oral mucosal epithelial cells, limbal epithelial cells, angiogenesis-related genes, gene expression, inflammation regulatory genes

## Abstract

Bilateral limbal stem cell deficiency (LSCD) can be effectively treated with cultivated oral mucosa epithelial cell transplantation (COMET). However, COMET is associated with greater superficial neovascularization than limbal stem cell (LESC) transplantation, the gold standard for unilateral LSCD. To investigate the intrinsic molecular features of cells intended for grafting, we assessed the in vitro expression of genes involved in vascularization and inflammation using real-time quantitative PCR and multifactorial linear models. Oral mucosal epithelial cells (OMECs) and limbal epithelial cells (LECs) were cultured in either conventional (COM) or xenobiotic-free (XF) media on fibrin substrates. Gene expression profiling revealed distinct transcriptional signatures. The pro-angiogenic genes *AGR2*, *ANGPTL2*, *CRYAB*, *EREG*, *JAM3*, and *S100A4* were significantly higher in LECs (adjusted *p* < 0.01), whereas *FGF2* was higher in OMECs (adjusted *p* < 0.001). The anti-angiogenic genes *TIMP3* and *SERPINF1* were higher in LECs (adjusted *p* < 0.01), while *COL18A1* was higher in OMECs (adjusted *p* < 0.01). OMECs also showed significantly greater expression of the immunoregulatory genes IL1B, IL6, TNF, CXCL10, and IL1RN (adjusted *p* < 0.01). Cultivation induced phenotypic changes in OMECs, with COM and XF media exerting comparable effects. These results highlight the contribution of inflammatory mediators to neovascularization following COMET.

## 1. Introduction

Limbal stem cell deficiency (LSCD) is an ocular disorder characterized by corneal vascularization, opacification, inflammation, and vision loss due to disruption of the limbal barrier [1]. While unilateral LSCD can be treated using ex vivo cultured limbal epithelial cells (LECs) from the contralateral eye [2], bilateral LSCD transplantation presents challenges due to the lack of autologous ocular stem cells. Cultivated oral mucosal epithelial transplantation (COMET) using autologous oral mucosal epithelial cells (OMECs) has emerged as an alternative [3,4].

COMET offers advantages such as cell availability, ease of cell harvesting, and low risk of immune rejection. Its success rate (~70.8%) is comparable to autologous LEC transplantation (~75%) [5]. However, COMET is frequently associated with peripheral corneal neovascularization, which develops to varying degrees in nearly all cases [3,4,6,7,8]. This neovascularization often progresses toward the central cornea within 3–6 months post operation before stabilizing at approximately one year. While this complication can impair visual function, it can be mitigated with immunosuppressive therapy [7].

Corneal avascularity is maintained by anti-angiogenic factors (e.g., pigment epithelium-derived factor, PEDF, encoded by *SERPINF1*; thrombospondin, THBS1; tissue inhibitor of metalloproteinase 3, TIMP3), while neovascularization is driven by pro-angiogenic mediators (e.g., vascular endothelial growth factor A, VEGFA; fibroblast growth factor 2, FGF2) [9].

Given the vascularized, pro-inflammatory nature of oral mucosa compared to the immune-privileged cornea [10,11,12], a detailed comparison of ex vivo LEC and OMEC sheet grafts is essential. Variations in experimental approaches and methodology affect angiogenic profiles, and the immunomodulatory properties of grafts remain unexplored [10,11,12,13,14]. This study examines the expression of vascularization- and inflammation-related genes in ex vivo LEC and OMEC sheets cultured on fibrin, comparing primary and cultured OMECs and evaluating conventional (COM) vs. xenobiotic-free (XF) culture conditions. Our choice of markers was guided by prior research comparing oral mucosal cells to limbal cells [10,11,12,13,14] as well as widely known indicators of inflammation [9].

We found that ex vivo cultured oral mucosal epithelial cells (OMECs) and LECs exhibit distinct angiogenic-related gene expression patterns, with OMECs showing higher *FGF2* and immunomodulatory gene transcription levels. Also, the culturing of OMECs significantly modified their angiogenic and immune profile, increasing the expression of *FGF2* and pro-inflammatory cytokines. Although conventional medium promoted earlier outgrowth and higher viability, the choice of culture medium (COM vs. XF) did not significantly impact gene expression patterns.

## 2. Materials and Methods

### 2.1. Donor Tissue

This study was conducted in accordance with the principles outlined in the Declaration of Helsinki. The collection of donor tissues complied with all legal requirements in the Czech Republic, including the condition that the donor will not be listed in the national register of individuals opposing the post-mortem removal of tissues and organs. The retrieval of corneoscleral and oral mucosal donor tissues was approved by the local ethical committee.

Eight cadaveric donor corneas (stored under hypothermic conditions) and eight cadaveric oral mucosal samples (6 mm round biopsy from the right and left buccal mucosa) were obtained from the Ocular Tissue Bank, Ophthalmology Department and Department of Pathology, University Hospital Kralovske Vinohrady, Prague, respectively. Specimens were prepared and stored as previously described [15,16]. For donor and tissue details, see Table S1 in Supplementary Material.

### 2.2. Cell Culture, Growth, and Viability

LECs were expanded as explant cultures [15], while OMECs were prepared from cell suspensions, according to the clinical standard [5,16]. Primary uncultured OMECs were stored for further analysis. Both cell types were cultured on fibrin-coated surfaces (Tisseel Lyo, Baxter, Zurich, Switzerland) in 12-well plates (300 µL fibrin/well), as previously described [15,16], in COM and XF media [15,17]. Cell growth was monitored using an Olympus CKX41 inverted phase-contrast microscope with an EOS 250D camera (Olympus CKX41; Olympus, Tokyo, Japan). Cultures were harvested at 85–95% confluency. The fibrin matrix was digested using 1.0 U/mL dispase II and TrypLE Express (Gibco, Paisley, UK) [18]. After washing and centrifugation (10 min at 250× *g*), a cell suspension (350,000 cells at least) was prepared and resuspended in 300 μL of RLT lysis buffer (RNeasy Micro Kit; Qiagen, Hilden, Germany) and stored at −80 °C until use. Cell viability and density were assessed using trypan blue staining (0.4%, Bio-Rad, Hercules, CA, USA) and a TC20 Automated Cell Counter (Bio-Rad, Hercules, CA, USA).

### 2.3. Cell Lysis and Real-Time Quantitative Polymerase Chain Reaction (RT-qPCR)

RNA was extracted using the RNeasy Micro Kit (Qiagen, Hilden, Germany) and diluted with RNase-free water. RNA quality and concentration were assessed via 260/280 and 260/230 nm ratios using an Eppendorf BioPhotometer (Eppendorf, Hamburg, Germany) and verified by agarose gel electrophoresis. Due to variability in RNA yield and quality from post-mortem oral mucosal tissue, only primary OMEC samples with sufficient RNA integrity and quantity were selected for gene expression analysis (*n* = 5 out of 8). This ensured consistency and reliability in downstream RT-qPCR assays. Samples were diluted to 25 ng/μL. cDNA was synthesized with the iScript cDNA Synthesis Kit (Bio-Rad, Hercules, CA, USA). RT-qPCR was performed in a 96-well plate using the SsoAdvanced Universal SYBR Green Supermix (Bio-Rad, Hercules, CA, USA) on a Bio-Rad CFX Connect system. Three technical replicates were conducted per sample. mRNA levels were normalized to *RPL32* and *HPRT1* using the ΔΔCt method [19]. Controls without templates and melting curve analysis ensured specificity. A complete list of the genes, their functions, and primer details are provided in Table 1 and Table S2.

### 2.4. Statistical Analysis

All statistical analyses were performed in R (v.4.4.0) [19]. Comparisons of growth dynamics and viability between LECs and OMECs were performed using *t*-tests or Wilcoxon rank-sum tests, depending on the normality (Shapiro–Wilk test, *p* < 0.05). Statistical significance was set at *p* < 0.05. RT-qPCR log-transformed data were plotted to explore their expression relationships using a hierarchical clustering heatmap and principal component analysis (PCA). Differential expression analysis was conducted using the limma package in R. The duplicate Correlation function was applied using donor identity as a blocking factor for OMECs. A linear model was then fitted to the data using lmFit, incorporating the estimated correlation. Differential expression was assessed through contrast comparisons, and empirical Bayes moderation was applied to improve variance estimation. Multiple testing correction was performed using the false discovery rate method. Adjusted *p* < 0.05 was considered as a threshold for statistical significance, with F-tests for multiple groups and *t*-tests for pairwise comparisons. Multifactorial analysis also accounted for age, sex, storage, and sample processing time. Data visualization was performed with the R package ggplot2 v.3.5.2. (https://cran.r-project.org/web/packages/ggplot2/index.html (accessed on 20 June 2025)).

## 3. Results

### 3.1. Cell Growth and Morphology

**Figure 1:**
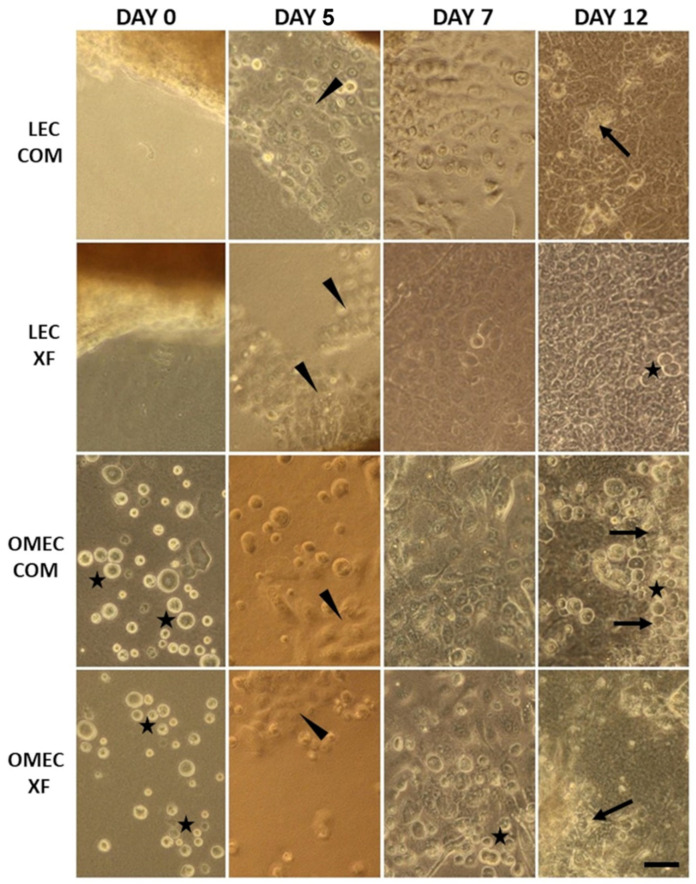
Inverted phase-contrast microscopy of cultured limbal epithelial cells (LECs) and oral mucosal epithelial cells (OMECs) on fibrin substrate in complex (COM) and xenobiotic-free (XF) media. The micrograph shows the progression of proliferation to confluence from the seeding day (0) to 5, 7, and 12 days of cultivation in representative images from different experiments. Note the round morphology of non-attached or proliferating cells (stars), the start of overgrowth from limbal explants or attached OMECs (arrowheads), and the stratification (arrows). Scale bar: 50 μm.

Limbal explant outgrowth was observed at 3.25 ± 0.89 (average ± SD) days in COM and 4.38 ± 1.06 days in XF conditions, with a significantly earlier onset in COM (paired *t*-test, *p* < 0.05) (Table S3). Similarly, in OMECs, cell attachment and proliferation began at 5.13 ± 0.83 days in COM and 5.75 ± 0.89 days in XF (Wilcoxon test, *p* < 0.05), again favoring COM. These findings indicate that COM supported faster initial growth kinetics than XF in both LECs and OMECs. Additionally, LECs initiated outgrowth earlier than OMECs (unpaired *t*-test, *p* < 0.05). The onset of outgrowth was characterized by a significant increase in adherent cell proliferation (Figure 1).

Despite these differences in initial growth, harvesting times were comparable across media and cell types, though viability tended to be higher in LECs than OMECs (Wilcoxon test, *p* < 0.05 for COM; unpaired *t*-test, *p* < 0.01 for XF). LECs reached 85–95% confluence after 10.88 ± 2.64 days in COM and 12.63 ± 2.26 days in XF (*p* > 0.05), with viability values of 92.5% ± 4.84 and 92.88% ± 5.18, respectively (*p* > 0.05). Immediately after seeding, OMECs displayed a rounded morphology, and by 7–10 days, they formed stratified sheets with a cobblestone-like morphology, prominent nuclei, and well-defined cell–cell contacts (Figure 1). OMECs reached 85–95% confluence in 11.25 ± 1.67 days in COM and 12.0 ± 1.85 days in XF (*p* > 0.05). Upon harvesting, OMEC viability was 84.76% ± 8.01 in COM and 79.76% ± 8.11 in XF, with a significant difference favoring COM (paired *t*-test, *p* < 0.05) (Table S3).

### 3.2. Angiogenic and Immunomodulatory Gene Expression

**Figure 2:**
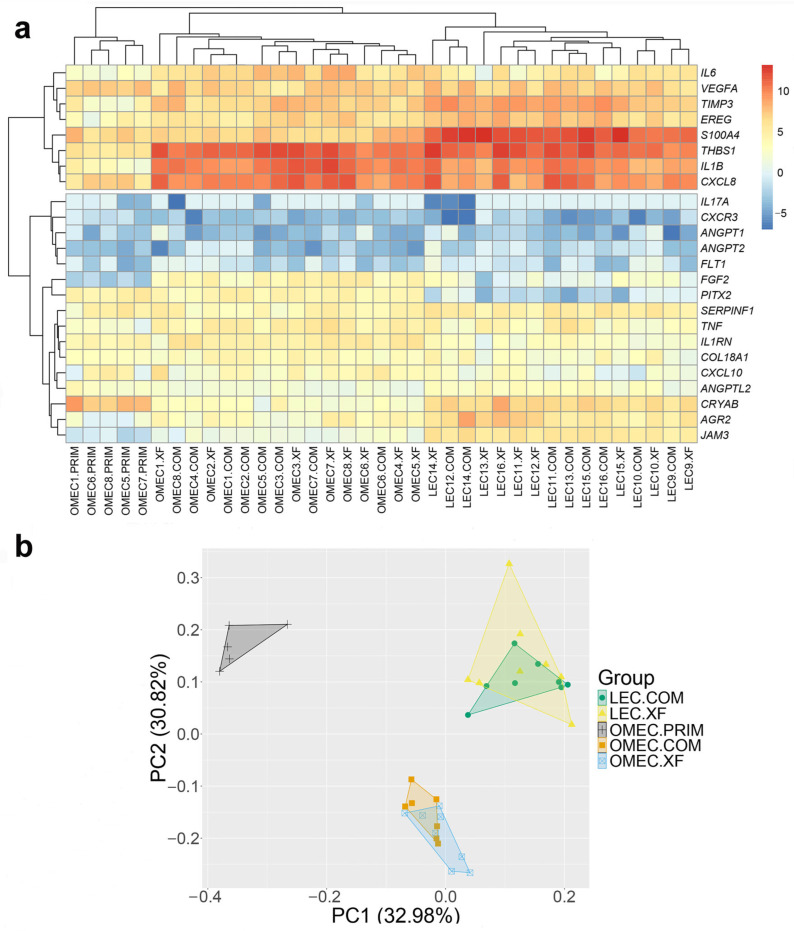
Angiogenesis- and inflammation-related gene expression profiles in limbal epithelial cells (LECs) and oral mucosal epithelial cells (OMECs). (**a**) Bidirectional hierarchical clustering heatmap of the relative mRNA expression for the tested genes (rows) between all samples (columns). The color scale on the right side of the plot represents higher expression in red and lower expression in blue. (**b**) Principal component analysis of the relative mRNA expression levels between all samples of LECs and OMECs. COM, complex medium; XF, xenobiotic-free medium; PRIM, primary culture of OMEC; the numbers on sample names refer to matched biological replicates.

The transcriptional profiles of genes regulating vascularization and inflammation were analyzed in LECs and OMECs by RT-qPCR. Bidirectional hierarchical clustering of relative mRNA expressions was used to identify natural groupings across both genes and samples, without imposing predefined categories. This analysis revealed two main sample categories: primary OMECs and cultured cells, with further clustering separating LECs and OMECs (Figure 2a). This outcome was independently confirmed by PCA, which identified three distinct clusters corresponding to primary OMECs, OMECs cultured in COM and XF media, and LECs cultured in COM and XF media. The first two PCA components explained 63.8% of the variance (Figure 2b).

**Figure 3:**
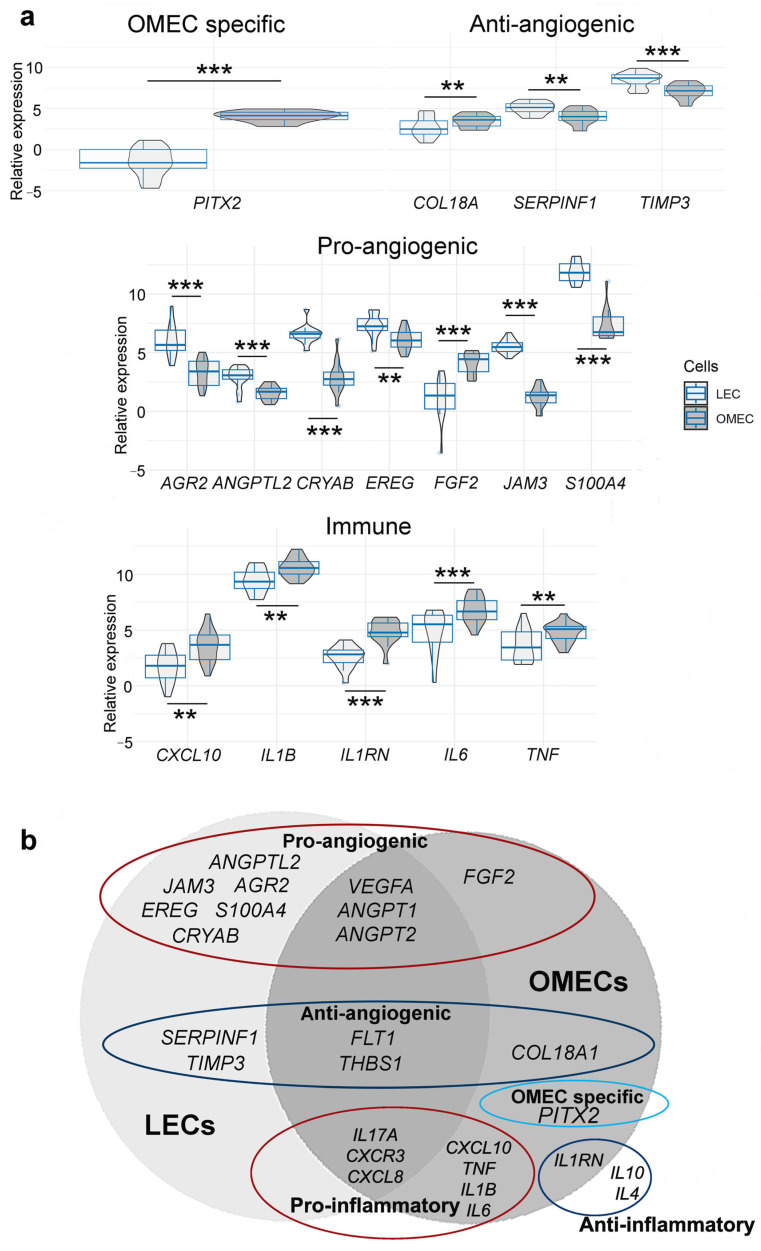
Differences and overlaps in selected angiogenesis- and immune-regulating genes between limbal (LECs, *n* = 16) and oral mucosal epithelial cell (OMECs, *n* = 16) cultures. (**a**) Statistically significant differences in the expression of *PITX2*, and angiogenesis- and immune-regulating genes between LECs and OMECs. Asterisks on the horizontal line above every two boxes indicate statistical significance defined as adj. *p* < 0.01 (**) and *p* < 0.001 (***). (**b**) Genes with higher or comparable expression in cultured LECs and OMECs, grouped according to their function.

No significant differences in gene expression were observed between COM and XF conditions in either LECs or OMECs (adj. *p* > 0.05 for all genes). Given the absence of significant media effects, gene expression comparisons were performed between LECs and OMECs, pooling COM and XF samples (Figure 3a). *PITX2* was preferentially expressed in OMECs (adj. *p* < 0.001). LECs had significantly higher expression of anti-angiogenic *SERPINF1* (adj. *p* < 0.01) and *TIMP3* (adj. *p* < 0.001), and also showed higher levels of pro-angiogenic *AGR2*, *ANGPTL2*, *CRYAB*, *JAM3*, *S100A4*, and *EREG* (adj. *p* < 0.001 for all except *EREG*, adj. *p* < 0.01). OMECs exhibited higher expressions of pro-angiogenic *FGF2* and anti-angiogenic *COL18A1* (adj. *p* < 0.001 for *FGF2*, adj. *p* < 0.05 for *COL18A1*). Additionally, OMECs demonstrated significantly higher expression of the pro-inflammatory cytokines *CXCL10*, *IL1B*, *IL6*, and *TNF* (adj. *p* < 0.01 to <0.001) and the anti-inflammatory *IL1RN* (adj. *p* < 0.001). The gene expression of the anti-inflammatory cytokines *IL4* and *IL10* was undetectable in either cell type. Both LECs and OMECs showed comparable expression of *VEGFA*, *ANGPT1*, *ANGPT2*, *FLT1*, and *THBS1*, as well as *CXCL3*, *CXCL8*, and *IL17A* (adj. *p* > 0.05, Figure 3b).

**Figure 4:**
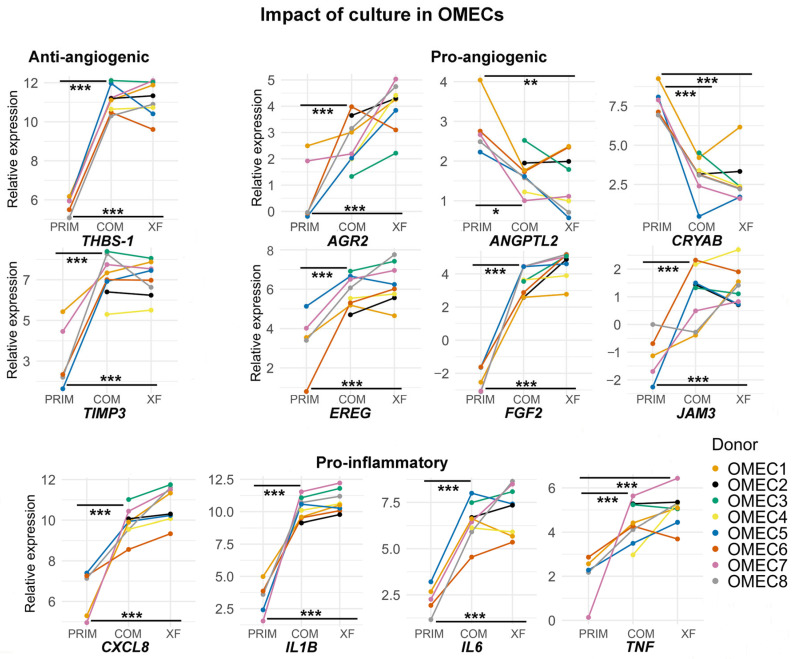
Significantly altered gene expression patterns of oral mucosal epithelial cells (OMECs) after culture. Color-coded lines connect data from the same donor. Expression of anti-angiogenic, pro-angiogenic, and immunomodulatory genes in primary cells before culture (PRIM, *n* = 5) and after culture in complex (COM, *n* = 8) or xenobiotic-free (XF, *n* = 8) medium. Asterisks indicate statistical significance defined as adj. *p* < 0.05 (*), *p* < 0.01 (**), and *p* < 0.001 (***).

Comparisons of primary uncultured and cultured OMECs (COM and XF) revealed significant changes in 12 genes (Figure 4). Notable findings included increased expression of the anti-angiogenic *THBS1* and *TIMP3* (adj. *p* < 0.001 for both COM and XF). Pro-angiogenic genes such as *AGR2*, *EREG*, *FGF2*, and *JAM3* were significantly upregulated in cultured OMECs (adj. *p* < 0.01 to <0.001). By contrast, there was a downregulation of *ANGPTL2* (adj. *p* < 0.05, COM; adj. *p* < 0.01, XF) and *CRYAB* (adj. *p* < 0.01, COM; adj. *p* < 0.001, XF). Pro-inflammatory cytokines, including *CXCL8*, *IL1B*, *IL6*, and *TNF*, were significantly elevated in cultured OMECs (adj. *p* < 0.001, COM and XF). There was a small positive within-donor correlation (correlation coefficient = 0.24) between primary and cultured OMECs.

## 4. Discussion

Neovascularization after COMET likely results from an imbalance of angiogenic factors [9]. We compared the angiogenic profile of ex vivo expanded LECs and OMECs [17,18], based on previously suggested markers [10,11,12,13,14], and investigated whether differences in immunoregulation contribute to this phenomenon.

With regard to anti-angiogenic gene expression, there is no consensus in previous studies, possibly due to diverged experimental models and culture conditions including media or cultivation surface. In our study, LECs and OMECs exhibited differential expression in three anti-angiogenic genes. LECs had higher *SERPINF1* (PEDF) and *TIMP3* mRNA levels, while *COL18A* (endostatin) was higher in OMECs. Interestingly, we did not find differences in the expression of *THBS1*, which is considered as the main anti-angiogenic factor maintaining corneal avascularity [14,41], and for which immunostaining was significantly higher in cultured LECs compared to OMECs [14]. We also found similar *FLT* mRNA levels in human ex vivo expanded LECs and OMECs, in accordance with a study from Sekiyama et al. (2006) comparing sFLT-1 immunoreactivity [14]. However, in the same study, PEDF and endostatin protein levels were all higher in LECs [14]. Using human biopsies, Chen et al. reported higher *TIMP3* but not PEDF immunoreactivity in corneal tissue compared to oral mucosa. In addition, healthy corneal tissues showed positive sFLT-1 immunoreactivity, which was absent in oral mucosa and conjunctival biopsies [10]. In vitro, rabbit LECs exhibited higher sFLT protein levels than OMECs, with recombinant sFLT-1 shown to reduce OMEC angiogenic capacity [12]. Therefore, the findings of this study regarding anti-angiogenic factors should be interpreted within the broader context of these existing discrepancies.

Unlike the inconsistencies observed in anti-angiogenic gene expression, all instances of pro-angiogenic factor expression aligned with previous studies. In our study, *VEGFA* and angiopoietins had similar levels in both cell types, consistent with previous reports [12,14]. Also, *AGR2*, *EREG*, and *JAM3* transcriptional levels were higher in LECs, as shown previously [13]. Although these genes are associated with angiogenesis and wound healing [13], their higher expression in LECs likely reflects physiological synthesis within the limbal niche [34], although their functions in the cornea may shift during inflammation or injury. A key finding was the significantly higher *FGF2* expression in the cultured OMECs than in the LECs, aligning with rabbit studies [12]. Moreover, in support of our findings, when LECs were used as feeder layers for OMECs, *FGF2* expression decreased, while *SERPINF1* levels were increased compared to OMECs grown on a 3T3 cell surface [54].

To our knowledge, a comprehensive comparison of immunomodulatory mediators between LECs and OMECs has not yet been conducted. We have shown that anti- and pro-vascularization genes are expressed in both cell types. However, OMECs show a significantly higher expression of immunomodulatory genes. It is well known that the angiogenic privilege of the cornea is closely tied to its immune privilege [55], and inflammatory mediators can increase FGF2 and VEGFA mRNA or protein levels [47,56]. Normal corneas maintain low pro-inflammatory cytokine expression, which rises under pathological conditions. For instance, reducing inflammation through cultivated limbal epithelium transplantation for LSCD lowers *IL1B* mRNA levels in the cornea to levels comparable to healthy tissue [46]. These findings raise the question of whether the higher expression of inflammation-related genes in the oral mucosa may be directly linked to an increased vascularization capacity of OMECs after grafting. Intrinsic differences, related to the immune and angiogenic phenotypes of OMECs, are possibly related to these findings. For example, in normal oral mucosal keratinocytes, similar cytokine expression patterns were found, without prior immune stimulation [57].

We have confirmed that the cultivation of OMECs significantly affected the expression of twelve genes, ten of which were upregulated. The simultaneous upregulation of both pro-angiogenic and anti-angiogenic factors likely reflects a complex adaptation of cultured OMECs to the in vitro environment. This upregulation may result not only from the activation of cell proliferation, as previously confirmed [17], but also from stress responses due to cultivation conditions or a feedback mechanism aimed at balancing angiogenic signaling. *CRYAB* and *ANGPTL2* downregulation may indicate a decline in pro-inflammatory or angiogenic signaling following in vitro expansion and highlight that not all pro-angiogenic genes follow the same pattern in response to cultivation. The observed reduction in *ANGLPTL2* expression, linked to inflammation and angiogenesis [22,23], was an unexpected finding, but it is still plausible that this change may be associated with the shift in the inflammatory phenotype observed in OMECs. Moreover, CRYAB gene downregulation may be associated with the regulation of inflammation [58], although the degree of this involvement merits further study.

In our experiments, we compared the growing efficiency of two media, COM and XF, using fibrin as a culture substrate [17]. Despite its potential to induce angiogenesis [59], we preferred this standardized substrate over the more biologically variable amniotic membrane. However, fibrin may still influence the angiogenic potential of both LECs and OMECs. Although harvesting times and final confluence levels were similar across media and cell types, COM significantly promoted earlier outgrowth and higher viability compared to XF, particularly in OMECs. This may indicate intrinsic differences in growth kinetics, possibly influenced by the culture protocol (explants versus primary cell suspensions). Nevertheless, gene expression differences between primary and cultured OMECs or LECs were unaffected by media choice.

The lack of a direct comparison between primary OMECs and LECs can be considered as a limitation of this study. LEC-based grafts are grown from explants [2], unlike OMECs, which are cultivated as suspension cultures [16]. For this reason, they were not included in our study. Another drawback is the use of different donors for OMECs and LECs, potentially introducing variability. Additionally, while these findings provide mechanistic insights, further validation at the protein level is essential.

Future research should aim to characterize OMEC-derived grafts in their final, transplantable form using both transcriptomic and proteomic analyses to determine whether these molecular signatures persist and how they influence clinical outcomes. Moreover, expanding beyond targeted gene panels to include unbiased, pathway-level approaches will be crucial for uncovering additional regulatory mechanisms and optimizing OMEC-based therapies while minimizing adverse events such as neovascularization. Although immunosuppressive strategies could help mitigate post-COMET neovascularization [3], reducing the need for postoperative medication use remains a priority due to the risk of adverse events.

## 5. Conclusions

In conclusion, ex vivo expanded OMECs and LECs exhibit distinct angiogenic and immunomodulatory gene expression profiles. Our most consistent finding across various models was the higher expression of *FGF2* and lower expression of *TIMP3* and *SERPINF1* in OMECs compared to LECs. This suggests a stronger angiogenic potential in OMECs, highlighting the critical role of these factors in regulating angiogenesis. One of the most notable findings was the significantly higher expression of immunomodulatory genes in cultured OMECs compared to cultured LECs, as well as their upregulation during cultivation. These transcriptional differences provide valuable insights into the functional characteristics of LECs and OMECs and their relevance for regenerative therapy in LSCD.

## Figures and Tables

**Table 1:** Gene functions and relevance to vascularization, inflammation, or oral mucosa identity.

Gene	Full Name	Function
Pro-angiogenic		
*FGF2*	Fibroblast growth factor 2	Promotes angiogenesis, wound healing, and cell proliferation [9,20,21]
*VEGFA*	Vascular endothelial growth factor A	Key driver of blood vessel formation under hypoxic or inflammatory conditions [9,20]
*ANGPTL2*	Angiopoietin like 2	Induces inflammation and neovascularization in tissue remodeling and cancer [22,23]
*ANGPT2*	Angiopoietin 2	Destabilizes blood vessels, facilitating VEGF-driven angiogenesis [24]
*ANGPT1*	Angiopoietin 1	Stabilizes blood vessels and supports endothelial cell survival [25]
*AGR2*	Anterior gradient 2, protein disulphide isomerase family member	Promotes epithelial cell growth and has roles in wound healing and tumor angiogenesis [26,27]
*CRYAB*	Crystallin alpha B	Acts as a molecular chaperone; involved in protection against stress and may support angiogenesis [28,29]
EREG	Epiregulin	EGFR ligand that promotes epithelial repair, proliferation, and angiogenesis [30,31]
S100A4	S100 calcium binding protein A4	Associated with motility, invasion, and angiogenesis in cancer and inflammation [32,33]
JAM3	Junctional adhesion molecule 3	Mediates cell–cell adhesion and contributes to angiogenesis [34,35]
Anti-angiogenic		
SERPINF1	Serpin family F member 1 (PEDF)	Potent anti-angiogenic factor that inhibits VEGF signaling [36,37]
FLT1	Fms related receptor tyrosine kinase 1 (VEGFR1)	Acts as a decoy receptor for VEGF, limiting angiogenesis [38]
TIMP3	TIMP metallopeptidase inhibitor 3	Inhibits matrix metalloproteinases and angiogenesis [39,40]
THBS1	Thrombospondin 1	Suppresses angiogenesis through interaction with CD36 and other receptors [14,41]
COL18A1	Collagen type XVIII alpha 1 chain	Encodes endostatin, a known inhibitor of angiogenesis [42]
Immuno-regulatory		
IL1RN	Interleukin 1 receptor antagonist	Anti-inflammatory cytokine that blocks IL-1 signaling [43]
CXCL10	C-X-C motif chemokine ligand 10	Chemokine that recruits immune cells and can inhibit angiogenesis [44]
CXCR3	C-X-C motif chemokine receptor 3	Receptor for CXCL9/10/11 involved in T-cell trafficking and inflammation [45]
IL1B	Interleukin 1 beta	Key pro-inflammatory cytokine promoting leukocyte recruitment [9,46]
IL6	Interleukin 6	Pro-inflammatory cytokine with roles in acute phase response and angiogenesis [9,47]
CXCL8	C-X-C motif chemokine ligand 8 (IL-8)	Promotes neutrophil chemotaxis and angiogenesis [9,48,49,50]
IL17A	Interleukin 17A	Stimulates pro-inflammatory responses and angiogenesis [51]
TNF	Tumor necrosis factor	Master regulator of inflammation, promotes angiogenesis under pathological conditions [9,50]
IL4	Interleukin 4	Anti-inflammatory cytokine, promotes Th2 immune response [52]
IL10	Interleukin 10	Anti-inflammatory cytokine that limits immune responses [53]
Oral mucosa identity		
PITX2	Paired like homeodomain 2	Transcription factor involved in oral epithelial identity and development [13]

## Data Availability

Data are available upon request to the corresponding author. The data are not publicly available due to ongoing analyses and related publications that are currently on preparation.

## Supplementary Materials

The following supporting information can be downloaded at https://www.mdpi.com/article/10.3390/bioengineering12070688/s1: Table S1. Details on the donors and samples. Table S2. Genes and primers used for RT-QPCR. Table S3. Growth parameters in limbal epithelial cells (LEC) and oral mucosal epithelial cells (OMEC).

## Abbreviations

The following abbreviations are used in this manuscript:

COM	Conventional culture medium
COMET	Oral mucosa epithelial cell transplantation
FGF2	Fibroblast growth factor 2
LECs	Limbal epithelial cells
LESC	Limbal epithelial stem cell
LSCD	Bilateral limbal stem cell deficiency
PCA	Principal component analysis
PEDF	Pigment epithelium-derived factor
RT-qPCR	Real-time quantitative polymerase chain reaction
THBS1	Thrombospondin
TIMP3	Tissue inhibitor of metalloproteinase 3
VEGFA	Vascular endothelial growth factor A
XF	Xenobiotic-free culture medium
